# Use of Tissue Culture Techniques for Producing Virus-Free Plant in Garlic and Their Identification through Real-Time PCR

**DOI:** 10.1155/2013/781282

**Published:** 2013-07-07

**Authors:** Hatıra Taşkın, Gökhan Baktemur, Mehmet Kurul, Saadet Büyükalaca

**Affiliations:** ^1^Department of Plant Production and Technologies, Faculty of Agricultural Sciences and Technologies, University of Niğde, 51240 Niğde, Turkey; ^2^Department of Biology, Faculty of Arts and Science, University of Osmaniye Korkut Ata, 80000 Osmaniye, Turkey; ^3^Roche Diagnostics, 34394 Istanbul, Turkey; ^4^Department of Horticulture, Faculty of Agriculture, University of Çukurova, 01330 Adana, Turkey

## Abstract

This study was performed for comparison of meristem culture technique with shoot tip culture technique for obtaining virus-free plant, comparison of micropropagation success of two different nutrient media, and determination of effectiveness of real-time PCR assay for the detection of viruses. Two different garlic species (*Allium sativum* and *Allium tuncelianum*) and two different nutrient media were used in this experiment. Results showed that Medium 2 was more successful compared to Medium 1 for both *A. tuncelianum* and *A. sativum* (Kastamonu garlic clone). *In vitro* plants obtained via meristem and shoot tip cultures were tested for determination of onion yellow dwarf virus (OYDV) and leek yellow stripe virus (LYSV) through real-time PCR assay. In garlic plants propagated via meristem culture, we could not detect any virus. OYDV and LYSV viruses were detected in plants obtained via shoot tip culture. OYDV virus was observed in amount of 80% and 73% of tested plants for *A. tuncelianum* and *A. sativum*, respectively. LYSV virus was found in amount of 67% of tested plants of *A. tuncelianum* and in amount of 87% of tested plants of *A. sativum* in this study.

## 1. Introduction

Total garlic production of Turkey was 105, 363 tons [1], and 18–20% of this production is provided from Taşköprü district of Kastamonu province. Kastamonu garlic clone (*A. sativum*) is suitable for winter consumption, can be stored for a long time, and is suitable for processing due to dry matter content [2]. *Allium tuncelianum*, called Tunceli garlic, is only common in Tunceli province of Turkey, especially around Munzur Mountains in Ovacık district, and it is known as an endemic to this region [3, 4]. Unlike other garlics with multiple-cloved bulb, *A. tuncelianum* has single-cloved bulbs and has small formations like a small bulbs, and it can also produce fertile flowers and seeds. The most important problem of garlic production in Turkey is infected areas due to pests and diseases carried via contaminated seedling. Garlic production areas and seedling production areas are not separated in Turkey. Pests and diseases can be carried via contaminated material in garlic propagated vegetatively because of sexual sterility. The most important ones of these diseases and pests are nematode (*Ditylenchus dipsaci*), white rot disease (*Sclerotium cepivorum*), and viruses that cause a loss of between 30% and 100% of production and the most common viruses are onion yellow dwarf virus (OYDV) and leek yellow stripe virus (LYSV). Fidan et al. [5] reported that these two viruses together affect the plant negatively and result in a yield loss of up to 78%. There is no effective chemical control method against viruses directly. For this reason, the most common method for obtaining virus-free plant is meristem culture technique. Different researchers [6, 7] have also reported that shoot tip remained insufficient for obtaining the virus-free plants. Biological (mechanical inoculation, indexing), serological (enzyme-linked immunosorbent assay—ELISA), and molecular (polymerase chain reaction—PCR) methods are used for detection of viruses in plants and identification of these viruses. Real-time 4 PCR assay, which is one of the PCR types, is able to provide quantitative assessment. One of the main advantages of the real-time PCR is the very low chances of infection and detection of viruses with high efficiency. Detection of viruses through real-time PCR method has been performed successfully by many researchers such as Roberts et al. [8], Korimbocus et al. [9], Balaji et al. [10], Lunello et al. [11], Yılmaz [12], Mehle et al. [13, 14], and Fidan et al. [5].

The objectives of this study were (i) comparison of meristem culture technique with shoot tip culture technique to obtain virus-free plants, (ii) comparison of micropropagation success of two different nutrient media and two different garlic species (*A. tuncelianum* and *A. sativum*), (ii) to see the effectiveness of real-time PCR assay on detection of viruses.

## 2. Material and Methods

This study was conducted at the Department of Horticulture, University of Çukurova Turkey, and Adana Veterinary Control Institute, Turkey between the years 2009 and 2010. *Allium sativum *(Kastamonu garlic clone) and *Allium tuncelianum* garlic species determined to be infected with viruses were used as plant material. *A. sativum* and *A. tuncelianum* garlic samples were provided by Kastamonu-Taşköprü and Tunceli-Ovacık regions of Turkey, respectively. 

### 2.1. Meristem and Shoot Tip Culture Studies

*A. sativum *and *A. tuncelianum* garlic species were propagated via meristem and shoot tip cultures. Cloves of garlic were waited in 25% sodium hypochlorite for 20 minutes for surface sterilization and washed 4-5 times with sterile water. Meristem and shoot tips from sterile cloves were extracted by sterile forceps and scalpels under a stereobinocular microscope in laminar flow and placed into glass tubes containing MS nutrient medium [15]. All cultures were incubated in the growth chamber at 25°C, under 3000 lux light and 8 hours dark and 16 h light photoperiod conditions. Germinated meristem and shoot tips were transferred to two different nutrient media (Medium 1: MS medium containing 0.5 mg L^−1^ 2-IP, 0.2 mg L^−1^ NAA, and 30 g L^−1^ sucrose; Medium 2: MS containing 2 mg L^−1^ BA, 0.5 mg L^−1^ IBA, and 30 g L^−1^ sucrose) for micropropagation. After 6 weeks, number of shoots per plant were recorded, and the shoots were subcultured. The experiment was designed in a completely randomized experimental design with three replications and thirty explants included per replication.

Variance analysis was conducted to evaluate the results, and Tukey test was used for controlling the significance of the differences.

### 2.2. Real-Time PCR Studies

Sixty *in vitro* plants (15 of them from meristem culture of *A. tuncelianum*, 15 of them from shoot tip culture of *A. tuncelianum*, 15 of them from meristem culture of *A. sativum*, and 15 of them from shoot tip culture of *A. sativum*) were used for real-time PCR studies. High Pure Viral Nucleid Acid Kit was used for RNA extraction of garlic samples. 200 *μ*L working solution and 50 *μ*L proteinase K were added to 1.5 mL nuclease-free microcentrifuge tube containing 100 mg plant samples and pulverized. After the mixture was mixed, it was incubated for 10 min at 72°C. Then, 100 *μ*L binding buffer was added and mixed. Mixture was transferred into the upper reservoir of combination of high pure filter tube and the collection tube and spun for 1 min at 8,000 g in a centrifuge. Collection tube was removed and filter tube was placed into a new collection tube. Then 500 *μ*L inhibitor removal buffer was added and spun for 1 min at 8,000 g in a centrifuge. Collection tube was changed with a new one. Then 500 *μ*L wash buffer was added and spun for 1 min at 10,000 g in a centrifuge. The collecting tube was changed, and the washing step was repeated. Filter tube was placed into new sterile 1.5 mL nuclease-free microcentrifuge tube, and 75 *μ*L elution buffer to elute the viral nucleic acid followed by spinning for 1 min at 13,000 g in a centrifuge. Filter tubes placed in the Eppendorf tubes were removed, and purified viral nucleic acid was extracted. 

Obtained pure viral nucleic acids were converted to cDNA with reverse transcription PCR (RT-PCR) and incubated at −15/−20°C in a freezer. Transcriptor High Fidelity cDNA Synthesis Kit was used for converting RNA to cDNA. This process was completed in two steps. 2 *μ*L random primer, 1 *μ*L PCR-grade water, and 8.4 *μ*L RNA per sample were used to prepare PCR mixture in the first step. This mixture was incubated at 65°C for 10 min. Prepared mixture in the second step consisted of 4 *μ*L transcriptor reverse transcriptase reaction tampon, 0.5 *μ*L protector RNAse inhibitor, 2 *μ*L deoxynucleotide mix, 1 *μ*L DTT, and 1.1 *μ*L transcriptor reverse transcriptase enzyme. These two mixtures were mixed and incubated at 45–55°C for 10–30 min and then at 85°C for 5 min. Thereafter, cDNA synthesis was completed and stored at −20°C. Real-time PCR mix-conducted in a 20 *μ*L volume containing 1xSYBR Green master mix (Roche applied science), 5 nM forward-reverse primers, and PCR grade water, using the following program: 10 min at 95°C for activation of DNA polymerase, 45 cycles of 10 s at 95°C, 30 s at 60°C, 1 s at 72°C, 30 min at 40°C and 30 s at 40°C for cooling in Roche LightCycler 2.0 Real-time PCR. Evaluation was performed by qualitative detection program of this thermocycler. Mixture containing 5 *μ*L isolated DNA from samples, 2 *μ*L SYBER Green master (it consisted, fast start Taq DNA polymerase, reaction buffer, MgCl_2_, dNTP mix, and SYBER Green-Roche), 5 *μ*L forward and reverse primers, 2.4 mM Mg, and PCR-grade water (total volume 20 *μ*L) was prepared for the detection in real-time PCR using 600 s at 95°C for denaturation of DNA, 45 cycles of 10 s at 95°C, 30 s at 60°C, 2 min at 72°C, and 30 min at 40°C for cooling. 

SYBER Green was used for detection of OYDV and LYSV viruses. The primers used in this study (Table 1) for OYDV and LYSV viruses were performed using primers registered in genbank [11] as forward OYDV primer (F-OYDV), 1099–1122 positions of sequences registered as AJ292223 in GenBank Database, as reverse OYDV primer (R-OYDV) 1306–1325 positions of AJ292223, as forward LYSV primer (F-LYSV) 789–808 positions of sequences registered as AY007693, and as reverse LYSV primer (R-LYSV) 839–854 positions of AY007693 [11].

## 3. Results

Ninety shoot tips and ninety meristems were used for *A. tuncelianum* in this experiment. Meristems transformed to plants in eight weeks. While 85 of meristems could grow without any problems, three of them could not germinate, and as infection problem was observed in two meristems. Cultured shoot tips were turned into plants in 3-4 weeks. Eighty-four shoot tips germinated healthy; only 1 shoot tip could not grow and 5 of ninety shoot tips were found to be infected. Plants obtained meristems of *A. tuncelianum* were transferred to two different nutrient media for micropropagation. Number of shoots were recorded in both first micropropagation and subculture were higher in Medium 2 compared to Medium 1, and the differences between the two media were found to be significant statistically. In the first propagation, 14.10 shoots/plant and 4.63 shoots/plant were obtained from Medium 2 and Medium 1, respectively. Similar results were provided from subculture. 13.27 shoots/plant in Medium 2 and 3.93 shoots/plant in Medium 1 were determined. Shoot tip culture results showed that Medium 2 was better compared to Medium 1. 11.37 shoots/plant and 2.41 shoots/plant were obtained from Medium 2 and Medium 1 in the first propagation, respectively. 8.93 shoots/plant and 2.36 shoots/plant were observed in Medium 2 and Medium 1 in subculture, respectively. Considered in terms of explants, meristem explants were found to be more successful compared to shoot tip explants in both garlic species and nutrient media.

The experiment for *A. sativum* was designated with 90 meristems and 90 shoot tips. While 89 meristems could transform into plant in meristem culture (the remaining one meristem was probably damaged), 87 shoot tips could germinate in shoot tip culture (the remaining 3 plants were infected). Meristem and shoot tip culture results of *A. sativum* showed that Medium 2 was more successful compared to Medium 1. Number of shoots/plant obtained from different tissue culture techniques and different garlic species are given in Table 2. As seen in Table 2, 11.67 shoots/plant and 5.41 shoots/plant were counted in Medium 2 and Medium 1 in the first micropropagation, respectively. In subculture, 11.61 shoots/plant and 5.56 shoots/plant were obtained on Medium 2 and Medium 1, respectively. These results compared with results of *A. tuncelianum*; number of shoots of *A. tuncelianum* in Medium 2 were higher than of *A. sativum* in both nutrient media. In Medium 1, *A. sativum* gave slightly higher results compared to *A. tuncelianum*. In shoot tip culture of *A. sativum*, Medium 2 and Medium 1 gave 9.84 shoots/plant and 2.93 shoots/plant for the first micropropagation, respectively. 10.17 shoots/plant from Medium 2 and 3.61 shoots/plant from Medium 1 were obtained in subculture.

**Figure 1:**
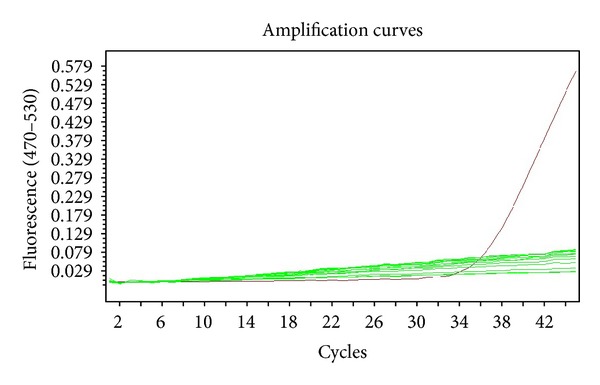
Application curves for meristem culture.

**Figure 2:**
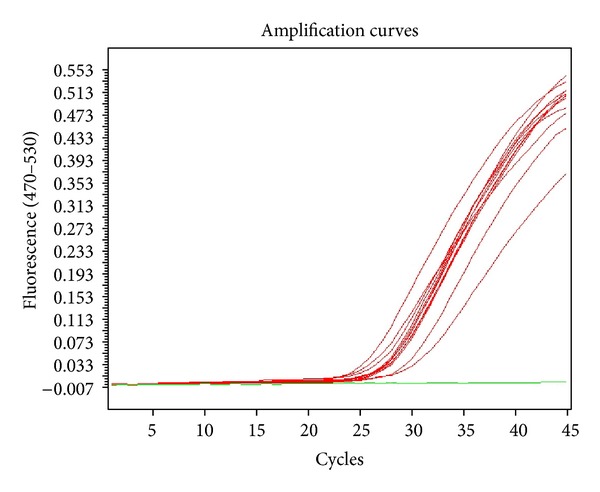
Application curves for shoot tip culture.

Sixty garlic plants propagated via meristem and shoot tip cultures were tested in terms of OYDV and LYSV viruses through real-time PCR method. Specific primer sequences and specific DNA fragments replicated with these sequences were detected with SYBER Green marking system. OYDV and LYSV viruses were not observed in plants obtained from meristem culture of both *A. tuncelianum* and *A. sativum*. Viruses were detected in shoot tip culture. OYDV virus was determined in 12 plants of *A. tuncelianum* and 11 plants of *A. sativum*. Ten plants of *A. tuncelianum* and 13 plants of *A. sativum* were found to be contaminated with LYSV virus. While OYDV virus was observed in the amount of 80% of tested plants and the amount of 73% of tested plants for *A. tuncelianum* and *A. sativum*, respectively, LYSV virus was found in the amount of 67% of tested plants of *A. tuncelianum* and the amount of 87% of tested plants of *A. sativum* in this study. Amplification curves of meristem and shoot tip cultures are given in Figures 1 and 2. 

## 4. Discussion

Results of this research showed clearly that meristem culture technique was more effective compared to shoot tip culture technique in obtaining virus-free plant. All plantlets propagated by meristem culture technique were found completely clear in terms of OYDV and LYSV. Whereas virus problem could not be solved by shoot tip culture technique, viruses were detected in the majority of plantlets obtained by this technique. In meristem culture, only apical dome and a few leaf primordia are isolated and placed to nutrient media [16]. Tips of growing shoots (2 cm or less than 2 cm) are used in shoot tip culture. According to some researchers, there is a competition between cell proliferation, and the formation of the virus particles in meristem region of plant. Nucleic acid production capacity in meristematic tissue during cell division is used for cell division and this situation prevents the reproduction of virus. According to other researchers, transportation of viruses to the meristem region of the plant is prevented due to lack of transport system in meristem [17].

ELISA and real-time PCR techniques are used commonly in diagnostics of plant viruses. Real-time PCR method is more sensitive and specific than other techniques. Therefore, recently researchers prefer to use this technique alone or as comparison with ELISA. Many researchers have used real-time PCR method to identify viruses in different plants: for Plum Pox virus in plum by Varga and James [18], for OYDV and LYSV viruses in garlic by Lunello et al. [11], for zucchini yellow mosaic virus (ZYMV) in squash by Çalışkan [19], and for OYDV and SLV viruses in onion and garlic by Fidan et al. [5]. Real-time PCR method was found to be more sensitive than other methods in diagnosis of potato virus Y (PVY) by Mehle et al. [14]. Therefore, real-time PCR method was used in this study to detect viruses in garlic plants. 

Also two different nutrient media were tested in terms of micropropagation success. Positive effect of BA and IBA on micropropagation of garlic was determined by Baktır [20]. NAA and 2-IP were used for *in vitro* propagation of garlic by Bhojwani [6]. Therefore, effects of these hormones on micropropagation of garlic were tested through using two different nutrient media in this study. Medium 2 containing 2 mg L^−1^ BA and 0.5 mg L^−1^ IBA was found to be more successful compared to Medium 1 containing 0.5 mg L^−1^ 2-IP and 0.2 mg L^−1^ NAA in this study.

One of the most important encountered problems in the production of garlic in Turkey is viruses. However, there is not any effective chemical application available against virus control. Meristem culture technique is widely used for obtaining virus-free plants. However, extracting the meristem regions of the plant is difficult, time-consuming and requires expertise. Therefore after achieving certain number of meristem, plants obtained from these meristems are multiplied using tissue culture techniques for commercial production. In this study, micropropagation capacity of *A. tuncelianum,* an endemic species of Turkey, and Kastamonu garlic clone of *A. sativum* were evaluated. The results obtained from the present study are very important for the scientist trying to develop virus-free plants and could also have significance of practical application which will in turn affect positively to increase yield with better quality of crop for the local farmers.

## Figures and Tables

**Table 1:** Primers used in the study [11].

Primer		bp
Forward-LYSV	5-TCTCGCACGGTATGCATTTG-3	20
Reverse-LYSV	5-GCCTCGCGCGCTCTAA-3	16
Forward-OYDV	5-AGTGATGCAGCTGAAGCATACATT-3	24
Reverse-OYDV	5-ACGTTACCATCCAGGCCAAA-3	20

**Table 2:** Number of shoots/plant obtained from different tissue culture techniques on different garlic species.

Explant Type	First micropropagation (shoots/plant)			Sub-culture (shoots/plant)		
	Medium 1	Medium 2	LSD (5%)	Medium 1	Medium 2	LSD (5%)
Meristem culture (AT)	4.63^b^	14.10^a^	0.86	3.93^b^	13.27^a^	1.86
Shoot tip culture (AT)	2.41^b^	11.37^a^	1.73	2.36^b^	8.93^a^	2.16
Meristem culture (AS)	5.41^b^	11.67^a^	1.21	5.56^b^	11.61^a^	2.58
Shoot tip culture (AS)	2.98^b^	9.84^a^	1.73	3.61^b^	10.17^a^	2.16

AT: *A. tuncelianum*; AS: *A. sativum.*

^a,b^Means followed by different letters in the same column are significantly different regarding Tukey's multiple range test at *P* < 0.05.
